# Plum-blossom needle for coronavirus disease 2019-related headache: A protocol for systematic review and meta-analysis

**DOI:** 10.1097/MD.0000000000022179

**Published:** 2020-09-11

**Authors:** Wei Gao, Jingfei Li, Guoxiang Huang

**Affiliations:** Encephalopathy Department, Chengdu Pidu District Hospital of TCM, China.

**Keywords:** coronavirus disease 2019, plum-blossom needle, systematic review

## Abstract

**Background:**

Assessing the effectiveness and safety of plum-blossom needle for (COVID-19) related headache is the main purpose of this systematic review protocol.

**Methods:**

We will search the following sources for the identification of trials: The Cochrane Library, PubMed, EMBASE, Chinese Biomedical Literature Database (CBM), Chinese National Knowledge Infrastructure Database (CNKI), Chinese Science and Technique Journals Database (VIP), and the Wanfang Database. The searches were limited to articles published in 2020, but no language restrictions were imposed. Only include randomised controlled trials (RCTs), with or without blinding, and participant or observer reported outcomes, will be included.

The primary outcome is the time and rate of appearance of headache induced by COVID-19. The secondary outcome is the length of hospital stay. Two independent reviewers will conduct the study selection, data extraction and assessment. Review Manager Software V.5.3 will be used for the assessment of risk of bias and data synthesis.

**Results:**

The results will provide a high-quality synthesis of current evidence for researchers in this subject area.

**Conclusion:**

The conclusion of our study will provide an evidence to judge whether plum-blossom needle is effective and safe for COVID-19-related headache.

**Ethics and dissemination:**

This protocol will not evaluate individual patient information or affect patient rights and therefore does not require ethical approval. Results from this review will be disseminated through peer-reviewed journals and conference reports.

**PROSPERO registration number:**

CRD42020199508.

## 1 Introduction

Coronavirus disease 2019 is a contagious disease with a rapid increase in cases and deaths since its first identification in Wuhan, China, in December 2019.^[1]^ SARA-CoV-2, a new kind of coronavirus and previously known as 2019-nCoV, contributed to the tragedy. This kind of virus is familiar with the Middle East respiratory syndrome CoV and the severe acute respiratory syndrome CoV genetically.^[2]^ Coronavirus disease 2019 (COVID-19) is highly infectious and can lead to fatal comorbidities especially acute respiratory distress syndrome (ARDS),^[3]^ which is life-threatening. In addition, it will cause symptoms of nervous system, like headache.^[4]^ The spike of proteins of COVID-19 could dictate tissue tropism using the angiotensin-converting enzyme type 2 receptor, which can be found in nervous system tissue, to bind to cells. As a result of the fact that there is no specific therapy for this new virus, dealing with the patient symptomatically become the only choice for the clinicians.^[5]^

Traditional Chinese medicine(TCM), which originates from China thousand years ago, has been playing a significant role in the treatment of COVID-19.^[6]^ Of TCM, acupuncture is a very important intervention, and has been applied all over the world.^[7]^ A plenty of researches have shown that acupuncture, including plum-blossom needle,^[8]^ has the function of alleviating the pain,^[9]^ including headache.^[10]^

This review aims to systematically evaluate the effectiveness and safety of plum-blossom needle for COVID-19-related headache by including multiple clinical trials published over the past 10 years.

## 2 Methods and analysis

### 2.1 Study registration

This systematic review protocol was registered with PROSPERO 2019 (registration number: CRD42020199508). And the protocol report is in the base of the Preferred Reporting Items for Systematic Reviews and Meta-Analyses Protocols (PRISMA) declaration guidelines.^[11]^ The review will be performed in line with the PRISMA declaration guidelines.^[12]^

### 2.2 Inclusion criteria for study selection

#### 2.2.1 Type of study

All randomised controlled trials (RCTs) about Plum-blossom needle for COVID-19-related headache which were reported in English and Chinese will be included. Trials with 2-arm or 3-arm parallel design will be also included. Non-RCTs, quasi-RCTs, case series, reviews, animal studies and any study with a sample size of less than 10 participants will be excluded.

#### 2.2.2 Type of participant

Patients with headache induced by COVID-19, regardless of sex, age, race or educational and economic status, will be included in the review.

#### 2.2.3 Type of interventions

Experimental interventions include plum-blossom needle therapy. Control interventions would be western medicine therapy.

#### 2.2.4 Type of outcome measures

The primary outcome is the time and rate of appearance of headache. The secondary outcome is the length of hospital stay.

### 2.3 Search methods for identification of studies

#### 2.3.1 Electronic data sources

We will search the following sources for the identification of trials: The Cochrane Library, PubMed, EMBASE, Chinese Biomedical Literature Database (CBM), Chinese National Knowledge Infrastructure Database (CNKI), Chinese Science and Technique Journals Database (VIP), and the Wanfang Database. The searches were limited to articles published in 2020, but no language restrictions were imposed

#### 2.3.2 Searching other resources

The reference lists of potentially missing eligible studies will be scanned ant the relevant conference proceedings will be scanned as well.

### 2.4 Search strategy

The search strategy for PubMed is shown in Table 1. The following search keywords will be used: plum-blossom needle; headache (eg, “head paint” or “head pains” or “pain, head” or “pains, head” or “cephalodynia” or “cephalodynias” or “cranial pain” or “cranial pains” or “pain, cranial” or “pains, cranial” or “cephalalgia” or “cephalalgias” or “generalized headache” or “generalized headaches” or “ocular headache” or “headache, ocular” or “headaches, ocular” or “ocular headaches” or “orthostatic headache ” or “headache, orthostatic” or “headaches, orthostatic” or “orthostatic headaches” or “vertex headache” or “headache, vertex” or “headaches, vertex” or “vertex headaches” or “retro-ocular headache ” or “headache, retro-ocular” or “headaches, retro-ocular” or “retro ocular headache” or “retro-ocular headaches” or “sharp headache” or “headache, sharp” or “headaches, sharp” or “sharp headaches” or “throbbing headaches” or “headache, throbbing” or “headache, throbbing” or “throbbing headaches” or “unilateral headache” or “headache, unilateral” or “headaches, unilateral” or “unilateral headaches” or “hemicrania” or “bilateral headache” or “bilateral headaches” or “headache, bilateral” or “headaches, bilateral” or “periorbital headache” or “headache, periorbita” or “headaches, periorbita” or “periorbital headaches”); COVID-19 (eg, “2019-nCoV” or “Wuhan coronavirus” or “severe acute respiratory syndrome CoV -2” or “2019 novel coronavirus” or “COVID-19 virus” or “coronavirus disease 2019 virus” or “COVID19 virus” or “Wuhan seafood market pneumonia virus”); randomized controlled trial (eg, “randomized controlled trial” or “controlled clinical trial” or “random allocation” or “randomized” or “randomly” or “double-blind method” or “single-blind method” or “clinical trial”. The equivalent search keywords will be used in the Chinese databases.

**Table 1:**
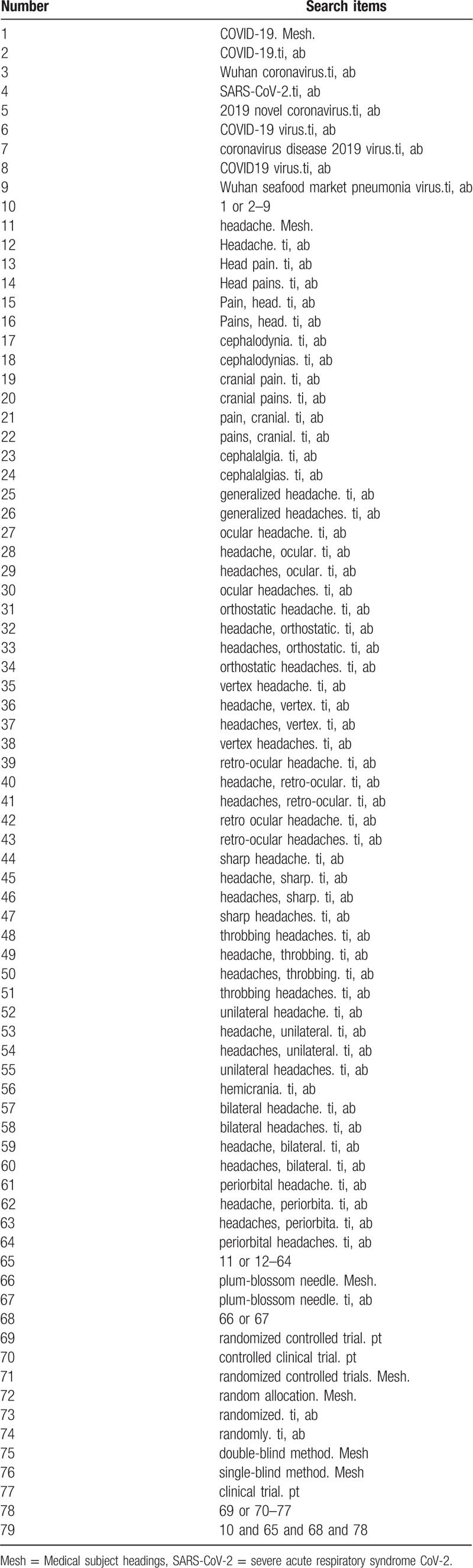
Search strategy for the PubMed database.

### 2.5 Data collection and analysis

#### 2.5.1 Selection of studies

**Figure 1:**
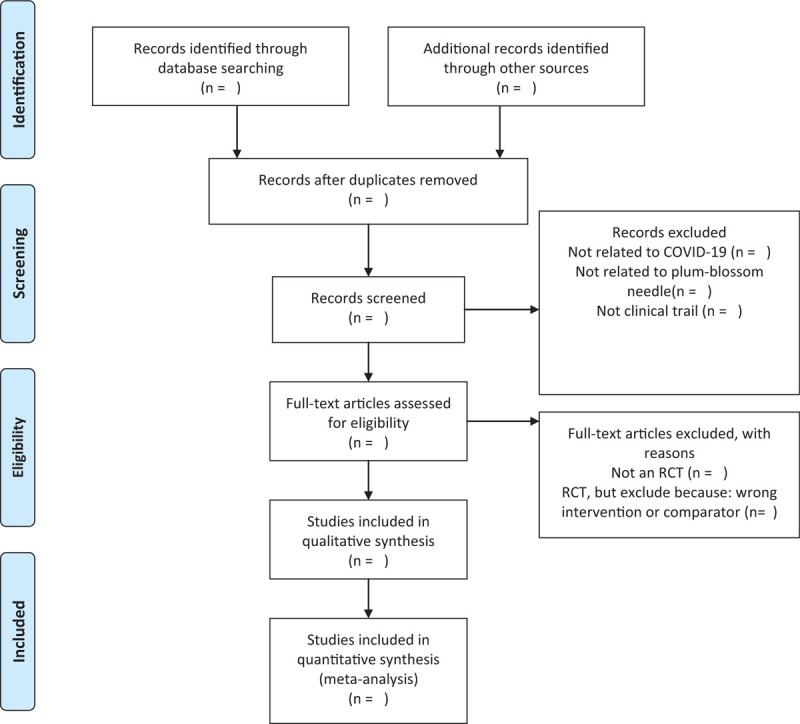
The PRISMA-P flow chart of the study selection process.

The titles and abstracts of all searched studies will be reviewed and screened independently by 2 reviewers, aiming at identifying eligible trials and eliminating duplicated or irrelevant studies in line with the criteria; the full text of all possibly eligible studies will obtained if necessary. A discussion with the third reviewer is planned to solve the disagreements. A PRISMA flow diagram will be used to show the study selection process in Figure 1.

#### 2.5.2 Data extraction and management

The following data will be extracted from the selected studies by 2 independent reviewers using a standard data extraction sheet: year of publication, country, general information, participant characteristics, inclusion and exclusion criteria, sample size, randomization, blinding methods, methods, control, outcome measures, results, adverse reactions, conflicts of interest, ethical approval, and other information.

#### 2.5.3 Assessment of risk of bias and reporting of study quality

Two independent reviewers will access the quality of included literature and complete the Standards for Reporting Interventions in Clinical Trials of Acupuncture checklist with the Cochrane collaboration risk-of-bias assessment method.^[13]^

#### 2.5.4 Measures of treatment effect

Dichotomous data will be presented as risk ratio and 95% confidence intervals, while continuous outcomes will be showed as standard mean difference 95% confidence intervals.

#### 2.5.5 Unit of analysis issues

The individual participant will the analytical unit.

#### 2.5.6 Management of missing data

Finding the cause of the missing data will be the solution. And 1 of us will contact the authors if the cause is not found. This will be documented and the available data will be extracted and analyzed if the missing data cannot be obtained.

#### 2.5.7 Assessment of heterogeneity

*I*^2^ test will be used to quantified inconsistency and standard *χ*2 test will be used to detect statistical heterogeneity. Studies will be considered to have homogeneity if the *P* value exceeds .1 and the *I*^2^ value is less than 50%, and the fixed-effects model will be used. while studies will be considered to have significant statistic heterogeneity if the *P* value is less than .1 or the *I*^2^ value exceeds 50%, and subgroup analysis will be used to explore the possible cause. And the random-effects model will be applied If the heterogeneity is still important.

#### 2.5.8 Assessment of reporting bias

Funnel plots will be used to access the reporting biases if there are over 10 trials included in the meta-analysis.

### 2.6 Data synthesis

Review Manager Software V.53 will be used for data synthesis. The random-effects model will be used if the I^2^ value is no less than 50%. The fixed-effects model will be used if the heterogeneity tests show little statistical heterogeneity. If there is meaningful heterogeneity that cannot be explained by any assessment, meta-analysis will not be performed.

### 2.7 Subgroup analysis

Subgroup analysis will be performed to explain heterogeneity if possible. Factors such as different types of control interventions and different outcomes will be considered.

### 2.8 Sensitivity analysis

Sensitivity analysis will be conducted to test the robustness of the review conclusions if possible. The impacts of sample size, study design, methodological quality, and missing data will be evaluated.

### 2.9 Grading of evidence quality

This paper will use the evidence quality rating method to evaluate the results obtained from this analysis. GRADE is generally applied to a large amount of evidence. It has 4 evaluation levels, namely, high, medium, low, and very low. GRADE was used to evaluate the bias, inconsistencies, discontinuities, and inaccuracies of test results. In the context of the system review, quality reflects our confidence in the effectiveness of assessment.^[14]^

### 2.10 Ethics and dissemination

This protocol will not evaluate individual patient information or affect patient rights and therefore does not require ethical approval. Results from this review will be disseminated through peer-reviewed journals and conference reports.

## 3 Discussion

This systematic review will be the first to assess the effectiveness and safety of plum-blossom needle for COVID-19-related headache, and its results will address a gap in the literature. The review contains 4 sections: identification, study inclusion, data extraction, and data synthesis. This review will aid doctors in the decision-making process for treating patients with symptoms of headache of COVID-19, and will provide information for patients and health policy makers.

## Author contributions

WG and JFL mainly contributed to this manuscript and joint first authors. GXH obtained funding. WG drafted the protocol. JFL makes the search strategy. GXH will obtain copies of the studies and WG will screen the studies to be included. Data extraction from the studies will be done by WG and JFL. JFL will put the data into Review Manager Software. Analyses will be conducted by WG. WG will draft the final review. GXH will act as an arbiter in the study selection stage. All authors have read and approved the final manuscript.
